# Clinical advances in transcutaneous auricular vagus nerve stimulation for post-stroke disorders: state of the art and future perspectives

**DOI:** 10.3389/fneur.2025.1676727

**Published:** 2025-10-31

**Authors:** Jifei Sun, Xuefei Li, Yuan Zhou, Hongwei Liu, Chenjie Ma, Chunbo Hao, Shuqing Liu, Jingxue Zhao, Xiaojian Zhang, Xue Xiao

**Affiliations:** ^1^Shunyi Hospital, Beijing Hospital of Traditional Chinese Medicine, Beijing, China; ^2^Eye Hospital, China Academy of Chinese Medical Sciences, Beijing, China; ^3^Guang’anmen Hospital, China Academy of Chinese Medical Sciences, Beijing, China; ^4^Beijing Tsinghua Changgung Hospital, Beijing, China

**Keywords:** transcutaneous auricular vagus nerve stimulation, stroke rehabilitation, neuromodulation, neuroplasticity, non-invasive therapy

## Abstract

Transcutaneous auricular vagus nerve stimulation (taVNS) has emerged as a promising non-invasive neuromodulation therapy for post-stroke disorders. This review synthesizes current evidence on the clinical efficacy and underlying mechanisms of taVNS in addressing a spectrum of post-stroke disorders, including motor, sensory, neuropsychiatric, and cognitive impairments. By targeting the auricular branch of the vagus nerve, taVNS modulates central pathways involved in neuroplasticity, anti-inflammation, angiogenesis, and blood–brain barrier protection, offering a multifaceted approach to stroke rehabilitation. Clinical studies demonstrate its potential to enhance functional recovery and improve quality of life, supported by its favorable safety profile and patient compliance. However, challenges such as parameter standardization, mechanistic elucidation, and individualized protocols remain. Future research should focus on large-scale trials, mechanistic exploration, and technological innovations to optimize taVNS applications in stroke care.

## 1 Introduction

Stroke is an acute cerebrovascular syndrome characterized by sudden-onset, rapidly progressive focal or diffuse neurological deficits resulting from intracranial vascular pathology (1). Pathologically, it is categorized into two principal subtypes: ischemic stroke and intracerebral hemorrhage. Epidemiological studies indicate that from 1990 to 2021, there was a significant increase globally in the number of stroke cases, related deaths, and disabilities caused by stroke. The incidence of stroke rose by 70%, mortality increased by 44%, and disability rates climbed by 32%, severely affecting patients’ quality of life and work capacity (2–4). The evolving disease landscape has positioned neurological disorders as the predominant contributor to global disease burden, surpassing cardiovascular conditions (5). Post-stroke patients frequently experience persistent deficits across multiple domains including motor function, language, cognition, swallowing, and psychological health. These debilitating sequelae not only profoundly compromise patients’ quality of life but also generate substantial caregiver burden and socioeconomic strain (2). Consequently, developing comprehensive early diagnostic frameworks, refining acute intervention protocols, and implementing evidence-based secondary prevention measures have emerged as paramount objectives in contemporary cerebrovascular disease management.

While conventional rehabilitation approaches—including physical therapy, occupational therapy, and speech therapy—demonstrate moderate efficacy in functional recovery, their therapeutic benefits remain limited for patients with moderate-to-severe neurological impairments (6). Beyond these standard rehabilitation protocols, clinicians have explored adjunctive neuromodulation techniques such as transcranial magnetic stimulation (TMS), though their clinical application remains constrained by limited treatment options and stringent eligibility criteria (7). In this therapeutic landscape, vagus nerve stimulation (VNS) has emerged as a promising intervention, demonstrating multidimensional benefits in stroke management. Current evidence indicates that VNS not only improves cardiovascular regulation and enhances neurological recovery, but also effectively mitigates common post-stroke complications including mood disorders such as anxiety and depression (8, 9). However, the invasive nature of conventional VNS implantation carries inherent risks, with potential adverse effects ranging from voice alterations (dysphonia) and swallowing difficulties (dysphagia) to various surgical complications.

The vagus nerve (VN) serves as a critical bidirectional communication pathway between the central nervous system and autonomic nervous system, playing a pivotal role in neuromodulation (10). Anatomically, the VN exhibits a unique fiber composition of 80% afferent and 20% efferent fibers, which underlies its sensory-motor integration capacity. Specifically, the afferent system primarily conveys visceral and somatic sensory information to brainstem nuclei, with visceral afferents predominantly projecting to the nucleus tractus solitarius (NTS) in the caudal medulla, while special sensory fibers from auricular and pharyngeal regions mainly terminate in the spinal trigeminal nucleus (11, 12). The efferent fibers originate principally from the nucleus ambiguus and dorsal motor nucleus of the vagus (13, 14), with this precise nuclear localization providing the structural basis for its functional organization.

In recent years, transcutaneous auricular vagus nerve stimulation (taVNS) has emerged as a promising non-invasive neuromodulation technique, attracting growing interest in both clinical and research applications. Compared to conventional invasive VNS, taVNS offers significant advantages: by selectively stimulating the auricular branch of the vagus nerve (ABVN)—the only superficially accessible vagal branch—it can directly activate both the NTS and dorsal motor nucleus (DMN) in the medulla, thereby establishing a complete “peripheral stimulation–central response” neural reflex circuit (15, 16); additionally, its completely non-invasive nature not only improves patient compliance but also eliminates the risks associated with surgical implantation, making it a safer and more sustainable option for long-term neuromodulation therapy (17).

**Figure 1:**
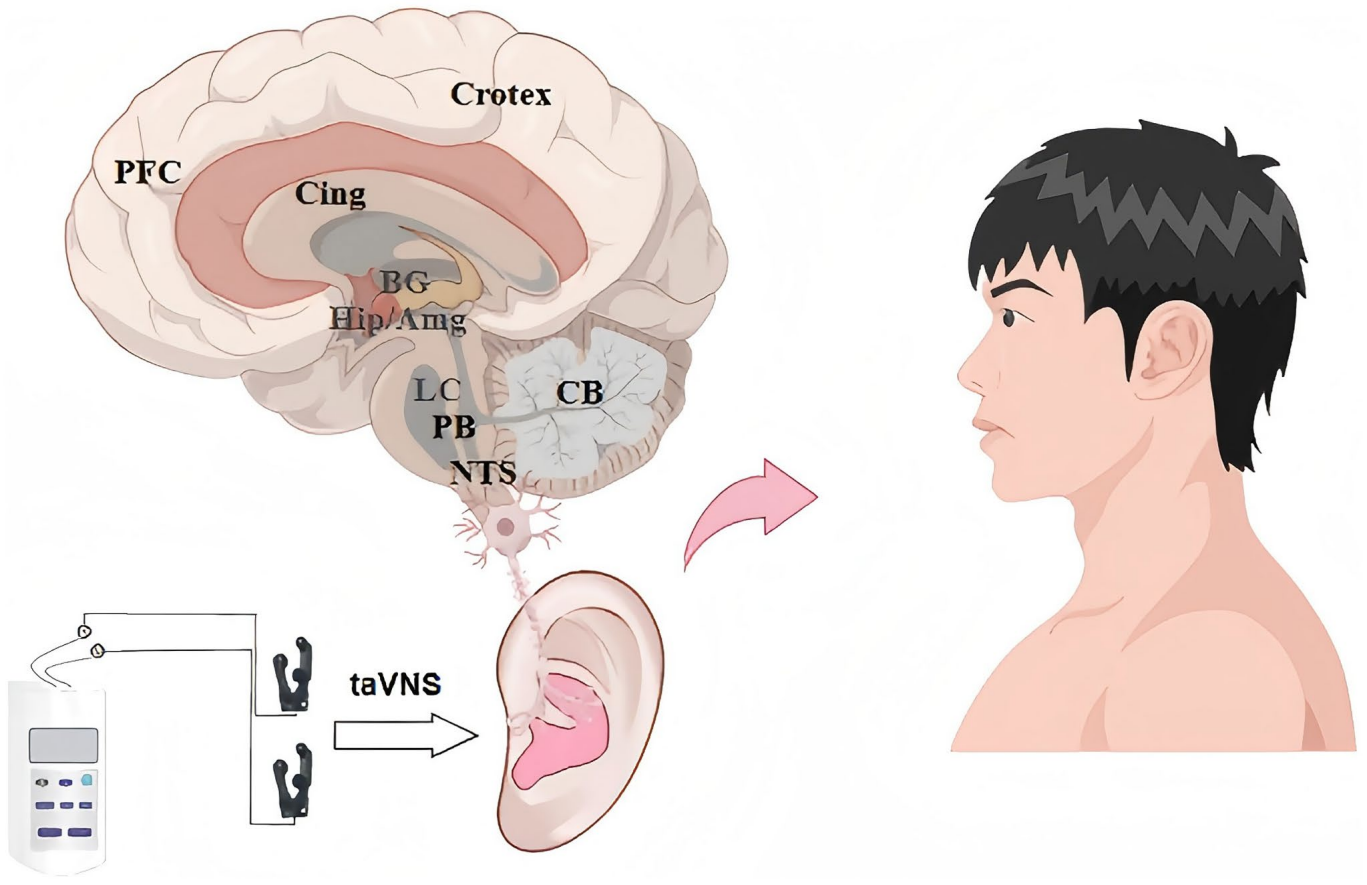
This figure illustrates the broad brain network activation triggered by taVNS, demonstrating its potential to modulate key regions involved in autonomic function, emotion, and cognition, which underpins its diverse therapeutic applications in stroke recovery. Key brain regions demonstrating significant activation include: the nucleus tractus solitarius (NTS), parabrachial nucleus (PB), locus coeruleus (LC), cerebellum (CB), hippocampus (Hip), amygdala (Amg), basal ganglia (BG), cingulate cortex (Cing), and prefrontal cortex (PFC).

The neuromodulatory effects of taVNS are mediated through a multilevel neural pathway (Figure 1): (1) primary afferent neurons relay stimulation signals to the NTS via synaptic transmission in the nodose ganglion (18); (2) as a key sensory integration center, the NTS distributes regulatory signals through its extensive projection network to multiple neuromodulatory nodes, including the brainstem reticular formation, hypothalamic autonomic centers, limbic emotional regulation circuits, and higher cortical functional areas (19); and (3) this hierarchical neural conduction mechanism enables precise modulation of autonomic nervous system function. Supported by robust mechanistic evidence and substantial clinical data, taVNS has demonstrated significant therapeutic potential in diverse applications, including ischemic cerebrovascular diseases and post-hemorrhagic neurological rehabilitation, positioning it as a novel interventional strategy for neurological disorders.

## 2 The clinical application of taVNS in stroke

**Figure 2:**
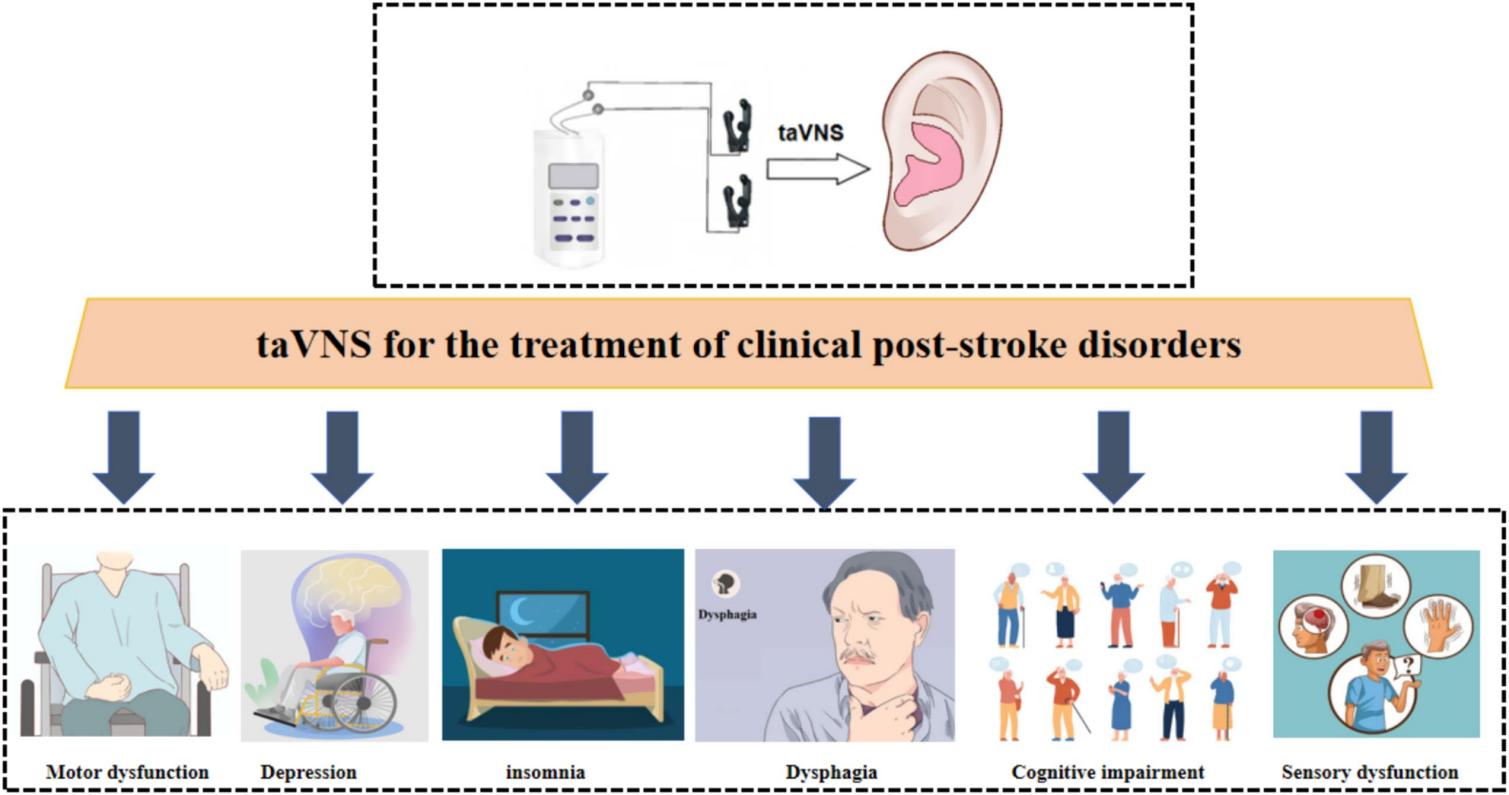
This figure summarizes various post-stroke functional deficits that have been explored in clinical studies and are potentially amenable to taVNS therapy, including motor dysfunction, depression, insomnia, dysphagia, cognitive impairment, and sensory dysfunction.

Recent years have witnessed increasing research interest in taVNS for stroke rehabilitation. Accumulating evidence suggests that taVNS exerts therapeutic effects on multiple post-stroke dysfunctions, including motor, sensory, swallowing, cognitive, and mood impairments, as well as sleep disturbances and disorders of consciousness. While the majority of studies have focused on taVNS for motor recovery, fewer investigations have examined its efficacy in addressing cognitive deficits and mood disorders (Figure 2). Notably, no studies to date have explored the potential role of taVNS in improving post-stroke speech function.

### 2.1 Therapeutic effects of taVNS on post-stroke limb dysfunction

Post-stroke limb motor dysfunction is a prevalent and debilitating condition that severely impacts patients’ functional independence and quality of life. Our systematic review of 13 clinical studies demonstrates strong evidence for the efficacy taVNS in motor rehabilitation (Table 1). Multiple randomized controlled trials utilizing sham-controlled designs (20–25) have confirmed the therapeutic benefits of taVNS, particularly when combined with conventional rehabilitation (23, 24, 26–28) or robotic training (21, 25). Notably, Li et al. (23) reported significantly greater motor improvement when taVNS was integrated with standard rehabilitation compared to control groups.

**Table 1:** Clinical studies of taVNS in stroke.

References	Population	Study design	group	Stimulation site	taVNS parameter	Main findings
Badran et al. (20)	Ischemic/hemorrhagic stroke, >6 months (*n* = 20)	Pilot RCT, Sham-controlled	taVNS+EMG sham taVNS+EMG	Left and right cymba conchae	Frequency 25 Hz Intensity 1–3 mA Pulse width 0.5 ms Stimulation duration 4 weeks	Improved and rebuild limb motor function
Chang et al. (21)	Chronic stroke, >6 months (*n* = 36)	RCT, Sham-controlled	taVNS+robot training sham taVNS+robot training	Left cymba conchae	Frequency of 30 Hz Intensity 0.1–0.5 mA Pulse width 0.3 ms Stimulation duration 3 weeks	Improved antagonistic motor function of the upper limb
Wang et al. (29)	Stroke, 14–45 days (*n* = 160)	RCT (No sham described)	taVNS+tDCS taVNS control group	Left ear	Frequency 25 Hz Pulse width 300 us Stimulation duration 4 weeks	Enhancing gait, balance, and activities of daily living
Wang et al. (22)	Stroke, >6 months (*n* = 40)	RCT, Sham-controlled	taVNS+TOT sham taVNS+TOT	Left auricular cymba concha	Frequency 25 Hz Pulse width 500 us Stimulation duration 4 weeks	Improved antagonistic motor function of the upper limb
Li et al. (23)	Stroke, >1 month (*n* = 60)	RCT, Sham-controlled	taVNS+CRT sham taVNS+CRT	Left and right ears	Frequency 30 Hz Pulse width 0.3 ms Stimulation duration 4 weeks	Improved upper limb motor and sensory dysfunction
Zhang et al. (26)	Stroke, 3–6 months post-onset (*n* = 124)	Non-randomized Clinical Trial	t-VNS + CRT	Left auricular cymba concha	Frequency 20/4 Hz Pulse width 0.2 ms Stimulation duration 4 weeks	Improved and rebuild limb motor function
Wang et al. (30)	Ischemic/hemorrhagic stroke, >3 weeks (*n* = 40)	Single-arm Pilot Study	taVNS	Left ear	Frequency 25 Hz Pulse width 300us Stimulation duration 30 min	Activation in the affected primary somatosensory cortex region following treatmen
Wu et al. (24)	Ischemic stroke, 0.5–3 months post-onset (*n* = 21)	Pilot RCT, Sham-controlled	taVNS+CRT sham taVNS+CRT	Left auricular branch vagus nerve	Frequency 20 Hz Pulse width 0.3 ms Stimulation duration 15 days	Improved antagonistic motor function of the upper limb
Capone et al. (25)	Ischemic/hemorrhagic stroke, >1 year (*n* = 14)	Pilot RCT, Sham-controlled	taVNS+robot sham taVNS+robot	Left auricular branch vagus nerve	Frequency 20 Hz pulse width 0.3 ms Stimulation duration 10 days	Improved and rebuild limb motor function
Redgrave et al. (27)	Stroke, >3 months post-onset (*n* = 13)	Open-label Pilot Study	taVNS+CRT	Left ear	Frequency 25 Hz Pulse width 1 ms Stimulation duration 6 weeks	Improved upper limb motor functions
Baig et al. (28)	Stroke, >3 months (*n* = 12)	Open-label Pilot Study	taVNS+CRT	Left ear	Frequency 25 Hz Pulse width 0.1 ms Stimulation duration 6 weeks	Improved upper limb motor functions
Liu et al. (31)	Stroke, >1 month (*n* = 80)	Double-blind RCT, Sham-controlled	taVNS+CRT sham taVNS+CRT	Left ear	Frequency 20 Hz Pulse width 0.3 ms Stimulation duration 6 months	Improved depressive symptoms
Zhao et al. (32)	Stroke, 7 months post-onset (*n* = 1)	Case Report	taVNS	Bilateral auricular concha areas	Frequency 20 Hz Pulse width 1 ms Stimulation duration 4 weeks	Improved insomnia symptoms
Wang et al. (35)	Stroke with swallowing problems, >2 weeks post-onset (*n* = 40)	RCT, Sham-controlled	taVNS+CRT sham taVNS+CRT	Left and right ears	Frequency 25 Hz Pulse width 0.5 ms Stimulation duration 4 weeks	Improved swallowing ability
Chen et al. (41)	Stroke, 2.5 years post-onset (*n* = 1)	Case Report	taVNS+CRT	Right ears	Frequency 20/4 Hz Stimulation duration 8 weeks	Improved cognitive impairment

TaVNS, transcutaneous auricular vagus nerve stimulation; CRT, conventional rehabilitation training; TOT, task oriented training.

The therapeutic window for taVNS intervention appears broad, spanning all phases of stroke recovery. Early intervention (14–45 days post-stroke) (29) through the subacute phase (1–6 months) (23, 24, 26) has shown promising results, with Wu et al. (24) reporting significant upper limb improvement in patients treated within 0.5–3 months. Importantly, taVNS maintains efficacy even in chronic stages (>6 months post-stroke), as demonstrated by Badran et al. (20) and Chang et al. (21), suggesting its potential as a long-term rehabilitation strategy.

Optimal stimulation parameters emerging from current research include medium frequencies (20–30 Hz) (20, 21), individualized current intensities (0.1–3 mA) (20, 21), and pulse widths of 0.2–0.5 ms (20, 21, 23–26). The left auricular site is predominantly used due to its direct vagal connections (21, 22, 24–31), though bilateral stimulation warrants further investigation (23). Treatment duration significantly impacts outcomes, with short-term protocols (≤2 weeks) (24, 25) showing initial benefits and extended regimens (4–6 weeks) (22, 27) producing more substantial improvements. Beyond motor recovery, taVNS enhances various functional domains including upper limb coordination (21), sensory restoration (19), cortical activation (30), and activities of daily living (29).

### 2.2 Therapeutic effects of taVNS on post-stroke depression

Liu et al. (31) recently conducted a double-blind, randomized, placebo-controlled trial to investigate the safety, efficacy, and potential molecular mechanisms of taVNS in treating patients with post-stroke depression (PSD). In this study, 80 patients diagnosed with PSD were enrolled. At the 6-month follow-up, the taVNS group demonstrated significantly greater reductions in HAMD-17 and SDS scores, along with improved Barthel Index (BI) scores, compared to the control group. Additionally, the taVNS group exhibited elevated serum levels of neurotrophic biomarkers suggesting modulation of the BDNF-CREB signaling pathway. Only minor transient adverse events, such as nausea, were reported, with no significant differences between groups. The findings indicate that taVNS combined with conventional treatment is a safe and effective intervention for alleviating depressive symptoms and enhancing functional recovery in PSD patients. Its non-invasive nature and minimal side effects make taVNS a promising therapeutic option for early-stage PSD management.

### 2.3 Therapeutic effects of taVNS on post-stroke insomnia

Stroke survivors with insomnia often experience poorer rehabilitation outcomes. A pioneering case report demonstrated that taVNS significantly improved sleep quality in a 64-year-old male patient with post-stroke insomnia (PSI) refractory to conventional drug therapy (32). This improvement may be attributed to taVNS-induced modulation of the default mode network (DMN) hyperconnectivity, a neural correlate of hyperarousal in insomnia. Following 4 weeks of self-administered taVNS (30 min twice daily), the patient’s Pittsburgh Sleep Quality Index score decreased from 13 to 8, with sustained effects observed at three-month follow-up. Resting-state functional magnetic resonance Imaging revealed reduced DMN connectivity in the posterior cingulate cortex (PCC) and enhanced functional coupling between PCC and visual/emotional processing regions. The authors proposed that taVNS may normalize hyperactive DMN activity while strengthening compensatory visual and emotional circuits, offering a portable, non-pharmacological intervention for PSI. However, these findings were derived from a single case.

The observed sleep improvements might be mediated by taVNS-driven rebalancing of thalamocortical circuits involved in sleep–wake regulation. Enhanced connectivity between PCC and visual cortices could reflect restored sensory gating, while strengthened thalamic integration may stabilize sleep-promoting pathways. Given the bidirectional relationship between DMN modulation and sleep quality, taVNS may initiate a positive feedback loop: reduced DMN hyperconnectivity alleviates hyperarousal, thereby facilitating deeper sleep, which further consolidates network normalization. These preliminary findings highlight taVNS as a potential catalyst for self-sustaining recovery in post-stroke sleep disorders.

### 2.4 Therapeutic effects of taVNS on post-stroke dysphagia

Post-stroke dysphagia, as a common and severe complication following stroke, affects 37–78% of patients and often leads to acute complications such as aspiration, pneumonia, and malnutrition, significantly impairing patients’ quality of life and functional recovery (33, 34). In recent years, taVNS has emerged as a novel neuromodulation technique demonstrating remarkable clinical potential in promoting neural functional remodeling. A randomized controlled trial conducted by Wang et al. (35) enrolled 40 patients with post-stroke dysphagia who were randomly assigned to either a taVNS group combined with conventional rehabilitation or a sham stimulation group. The treatment protocol consisted of 30 min sessions administered twice daily, five times per week for three consecutive weeks. Results showed that patients in the taVNS group exhibited significant improvements across multiple standardized assessment measures, including the Modified Mann Assessment of Swallowing Ability, Functional Communication Measures, and Rosenbek’s Penetration-Aspiration Scale, indicating the therapy’s efficacy in enhancing swallowing function. Notably, taVNS significantly improved tongue motility, enhanced cough reflex sensitivity, and strengthened soft palate contraction, supporting its therapeutic role in optimizing neuromuscular control mechanisms related to swallowing (35, 36). Particularly encouraging was the finding that the therapeutic effects persisted for at least 4 weeks post-intervention, demonstrating sustained clinical benefits.

The neuroprotective effects of taVNS likely involve multiple pathways. Primarily, this technique specifically activates brainstem nuclei, which constitute the central pattern generator for swallowing (15), thereby directly regulating pharyngeal and laryngeal muscle function (37). While current evidence confirms that taVNS improves cough reflex and soft palate function through enhanced laryngeal sensory feedback and motor control, its precise regulatory mechanism on salivary control requires further elucidation, particularly regarding its causal relationship with activation of the central pattern generator (38, 39). Although the exact mechanisms demand further investigation, existing evidence strongly suggests that taVNS may improve swallowing function by synergistically enhancing sensorimotor integration and optimizing reflex arc regulation mechanisms.

### 2.5 Therapeutic effects of taVNS on post-stroke cognitive impairment

Post-stroke cognitive impairment (PSCI) leads to deficits in memory, comprehension, perception, language, and executive function, consequently reducing quality of life, slowing functional recovery, and serving as an independent risk factor for increased mortality in stroke patients (40). A clinically instructive case study demonstrated that after 8 weeks of taVNS intervention in a 71-year-old patient with chronic PSCI, the Montreal Cognitive Assessment score improved by 57% (41), executive function test completion time decreased by nearly 50%, and diffusion tensor imaging confirmed significant improvement in fractional anisotropy within the bilateral dorsolateral prefrontal cortex (DLPFC). These objective findings indicate that home-administered taVNS not only alleviates clinical symptoms but also promotes white matter microstructural remodeling in cognition-related brain regions.

TaVNS may enhance structural-functional connectivity in key regions like the DLPFC through multiple mechanisms including neuroplasticity modulation, neuroinflammation suppression, and functional network reorganization, thereby improving cognitive function (42). Given its non-invasive nature and operational simplicity, taVNS shows promise as a groundbreaking approach for community-based PSCI management (41). However, current evidence remains limited to case studies. Future multicenter randomized controlled trials are warranted to establish optimal stimulation parameters and identify suitable patient populations, thereby providing evidence-based foundations for clinical guideline development.

### 2.6 Therapeutic effects of taVNS on post-stroke sensory dysfunction

Sensory dysfunction is one of the most serious complications following stroke. When patients experience impaired sensory function, it directly affects their motor control capabilities, thereby negatively impacting their most basic functional activities and severely compromising their daily living (43). Researchers recently conducted a pilot study to investigate the impact of taVNS paired with upper limb repetitive task practice on sensory recovery in chronic stroke patients (28). In this study, 12 participants who were more than 3 months post-ischemic stroke with residual upper limb weakness received 18 sessions of taVNS combined with motor rehabilitation over 6 weeks. Post-intervention assessments revealed that 64% of participants showed improvements in proprioception, while 25% exhibited enhanced light touch sensation, as measured by the Upper Limb Fugl-Meyer assessment. Notably, the participant with the greatest motor improvement also demonstrated the most significant sensory recovery. The intervention was well-tolerated, with no serious adverse events reported. These findings suggest that taVNS paired with motor rehabilitation may promote sensory recovery in chronic stroke patients, potentially through mechanisms involving neuroplasticity. The non-invasive nature and safety profile of taVNS highlight its promise as an adjunct therapy for sensory and motor rehabilitation in stroke survivors. Further phase 2 studies are warranted to validate these results and explore the underlying mechanisms.

## 3 The underlying mechanism of taVNS in stroke

**Figure 3:**
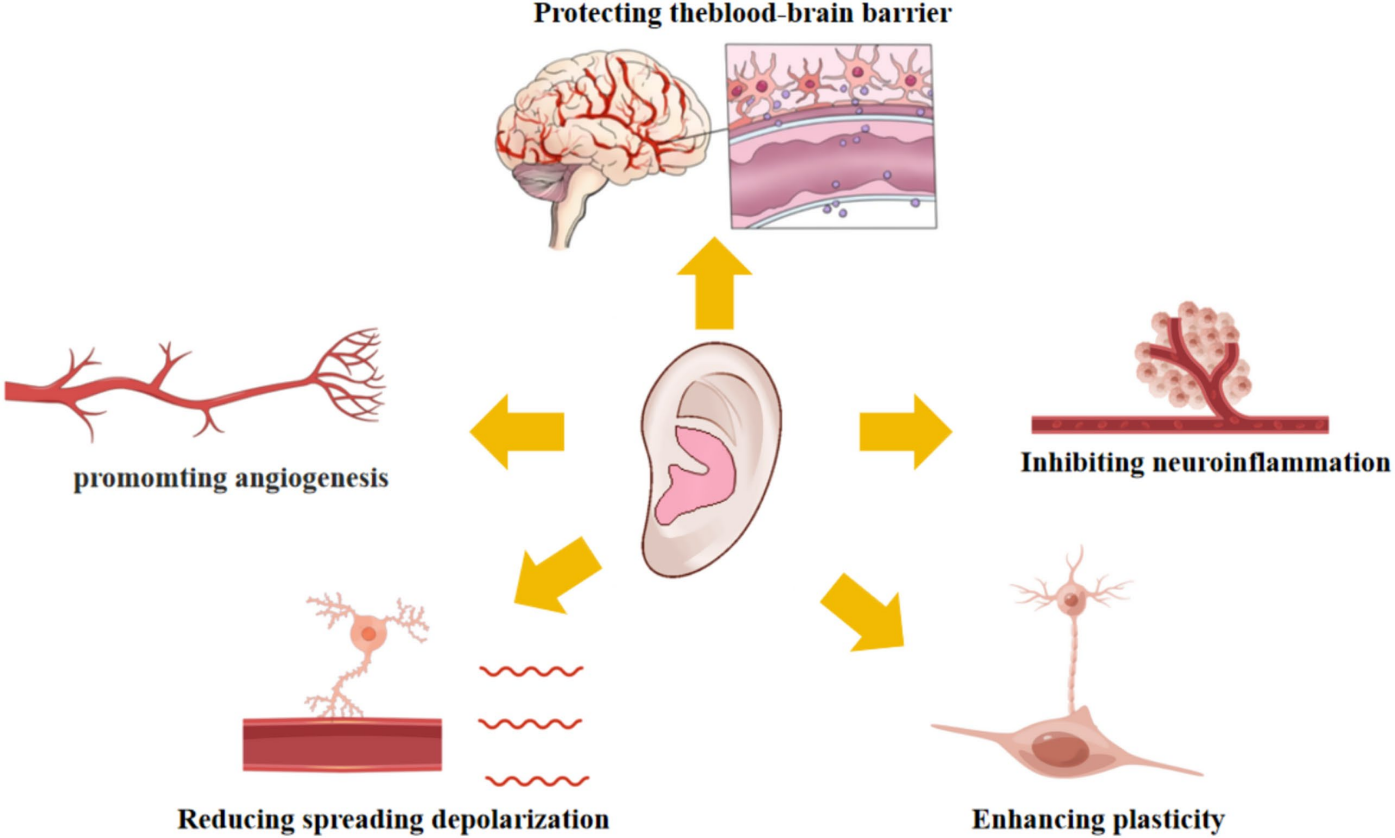
This figure provides a multi-mechanistic overview of how taVNS confers neuroprotection and promotes recovery at the cellular and molecular levels, including anti-inflammation, angiogenesis, and enhanced plasticity, as observed primarily in preclinical models.

TaVNS has demonstrated multifaceted neuroprotective and restorative effects in experimental models of stroke, primarily mediated through five interrelated mechanisms: anti-inflammatory properties, promotion of angiogenesis and neuroprotection, reduction of spreading depolarization, enhancement of neural plasticity, and preservation of blood–brain barrier integrity (Figure 3; Table 2). These mechanisms collectively contribute to functional recovery by modulating key pathophysiological processes post-stroke. The following sections detail each mechanism, synthesizing evidence from preclinical and clinical studies.

**Table 2:** The underlying mechanism of taVNS in stroke.

References	Rodent models	Device	Initial time	taVNS parameter	Stimulation site	Key Biomarkers/Effects	Results and conclusion
Zhao et al. (49)	Rat stroke model	taVNS	24 h post-ischemia	Frequency 10 Hz Intensity 1 mA Pulse width 0.5 ms Stimulation duration 7 days	Bilateral auricular concha	Pro-inflammatory cytokines; Connexin-43	Improved motor function via anti-inflammatory effects and gap junction modulation
Long et al. (54)	Rat MCAO model	taVNS	30 min post-occlusion	Frequency 20 Hz Intensity 0.5 mA Pulse width 0.5 ms Stimulation duration 28 days	Left cavum concha	PPAR-γ, BDNF, VEGF, p-eNOS; inflammatory markers	Enhanced functional recovery, reduced infarction, promoted angiogenesis
Gong et al. (56)	Mouse MCAO model	taVNS	2 h post-stroke	Frequency 20 Hz Intensity 1 mA Pulse width 330 ms Stimulation duration 7 days	Left auricular region	Ferroptosis markers; α7nAChR	Promoted neurogenesis, angiogenesis, and suppressed inflammation
Jiang et al. (57)	Rat MCAO model	taVNS	30 min post-stroke	Frequency 20 Hz Intensity 0.5 mA Pulse width 0.5 ms Stimulation duration 3 weeks	Left cavum concha	Neurotrophic factors; vascular markers	Reduced infarct volume, improved behavior, enhanced microvasculature
Li et al. (58)	Rat MCAO mode	taVNS	90 min post-stroke	Frequency 20 Hz Intensity 0.5 mA Pulse width 0.5 ms Stimulation duration 28 days	Left cavum concha	α7nAChR; BDNF/cAMP/PKA/p-CREB pathway	Neuroprotection via activation of plasticity-related signaling pathways

### 3.1 Anti-inflammatory property

TaVNS has demonstrated extensive applications in animal models of stroke, exhibiting multiple therapeutic effects including: anti-inflammatory properties, promotion of angiogenesis and neuroprotection, suppression of spreading depolarization, enhancement of neural plasticity, and preservation of blood–brain barrier integrity (Figure 3; Table 2). Existing studies have shown that the anti-inflammatory mechanisms of taVNS primarily involve the following three key pathways. First, the cholinergic anti-inflammatory pathway is the core mechanism by which taVNS exerts its anti-inflammatory effects (44). When the vagus nerve is stimulated by taVNS, the terminal release of acetylcholine (ACh) specifically binds to *α*7 nicotinic acetylcholine receptors (α7nAChR) on the surface of macrophages (45). This binding process significantly inhibits the activation of the NF-κB signaling pathway while modulating the JAK2/STAT3 signal transduction pathway, thereby effectively reducing the expression levels of key pro-inflammatory factors such as IL-1β and TNF-α (46, 47). Second, the hypothalamic–pituitary–adrenal axis plays a crucial role in the anti-inflammatory effects of taVNS (48). Excitatory stimulation of the vagus nerve activates specific neuronal populations in the hypothalamus, prompting the anterior pituitary to release adrenocorticotropic hormone (ACTH), which in turn stimulates the adrenal cortex to secrete glucocorticoids (49). These glucocorticoids exert broad inhibitory effects on various immune cell functions through both genomic and non-genomic mechanisms, thereby generating a systemic anti-inflammatory response (50). Third, the splenic sympathetic nerve anti-inflammatory pathway constitutes another important mechanism underlying the anti-inflammatory effects of taVNS. Upon vagus nerve excitation, sympathetic nerve activation is achieved through neural-neural synaptic transmission, promoting the release of norepinephrine (51).

The main features of neuroinflammation include the activation of reactive astrocytes and microglia. Activated microglia release large amounts of inflammatory cytokines, leading to reperfusion injury (52). Ischemic brain injury has been shown to persist and progress long after infarction. Based on the study by Zhao et al. (53), taVNS significantly reduces inflammatory cytokine levels in cerebral ischemia/reperfusion injury by activating the cholinergic anti-inflammatory pathway. Experimental results demonstrated that after 7 days of taVNS intervention, the secretion of pro-inflammatory cytokines (TNF-*α*, IL-1β, IL-6) in the ischemic penumbra and motor cortex of rats was markedly decreased, while ACh levels increased. ACh binds to α7nAChR on macrophages, inhibiting the NF-κB signaling pathway and Cx43 phosphorylation, thereby alleviating neuroinflammatory responses. Furthermore, taVNS exerts additional anti-inflammatory effects by modulating microglial polarization and suppressing NLRP3 inflammasome activation. Another study found that taVNS can significantly suppress inflammatory responses in the white matter following cerebral ischemia (54). Western blot and ELISA analyses revealed that the expression levels of pro-inflammatory factors IL-1β and TNF-α were markedly lower in the taVNS treatment group compared to the control group, along with reduced expression of key inflammatory signaling proteins such as TLR4, MyD88, phosphorylated MAPK, and NF-κB. These findings suggest that taVNS may alleviate post-ischemic neuroinflammation by modulating the TLR4/NF-κB and MAPK/NF-κB signaling pathways, thereby creating a favorable microenvironment for white matter repair.

### 3.2 Promoting angiogenesis and neuroprotection

Ischemic brain injury caused by insufficient cerebral blood perfusion is the core pathophysiological characteristic of ischemic stroke. Studies have demonstrated that cerebrovascular remodeling plays a crucial role throughout the entire process of post-stroke neurological recovery, in which the establishment of collateral circulation and the activation of angiogenesis serve as the primary mechanisms for improving cerebral blood perfusion (40).

A recent study investigated the therapeutic mechanism of taVNS in post-stroke recovery (55). The research revealed that in a mouse model of stroke, taVNS activates α7nAChR, leading to upregulation of GPX4 and downregulation of ACSL4 expression. Concurrently, this stimulation promoted neurogenesis and angiogenesis while reducing neuroinflammation. Another study demonstrated that taVNS significantly improved neurological deficit scores, balance capacity, and sensorimotor function (56). The therapeutic effects were mediated through upregulation of brain-derived neurotrophic factor (BDNF), phosphorylated endothelial nitric oxide synthase (p-eNOS), and vascular endothelial growth factor (VEGF) expression, which enhanced angiogenesis and endothelial cell proliferation in the ischemic penumbra. In addition, Long et al. (54) found that taVNS promotes angiogenesis in the white matter region by increasing the expression of VEGF and basic fibroblast growth factor (FGF2).

### 3.3 Reducing spreading depolarization

In the ischemic penumbra, the triggering of spreading depolarization (SD) is closely associated with multiple pathological factors, including post-ischemic energy metabolism dysfunction, oxidative stress, and inflammatory responses (57). Once initiated, recurrent SD events further compromise energy supply in the penumbra, leading to severe ATP depletion and consequently accelerating cellular apoptosis and necrosis (58). Animal model studies have confirmed that suppressing SD significantly reduces infarct volume, strongly suggesting SD plays a pivotal role in the expansion of cerebral infarction (59). These findings collectively indicate that SD not only serves as a critical mediator of ischemic penumbra injury but also represents a potential therapeutic target for intervening in stroke progression.

### 3.4 Enhancing brain plasticity

The neural network’s physiological functions are fundamentally based on synaptic connections between neurons that mediate information transmission. Ischemic injury compromises synaptic structural integrity, disrupting signal transmission and resulting in sensorimotor dysfunction (60, 61). As synapses serve as functional units for neurotransmitters and receptors, they constitute the basic structural foundation for intercellular signaling throughout the central nervous system (62). Changes in synaptic ultrastructure largely reflect the plasticity of the nervous system and represent the neurobiological basis of cerebral plasticity following ischemia–reperfusion injury.

One study demonstrated that taVNS enhances post-stroke axonal plasticity by activating α7nAchR receptors (63). Specifically, taVNS upregulates α7nAchR expression, which in turn activates the BDNF/cAMP/PKA/p-CREB pathway, thereby promoting axonal regeneration and reorganization to improve neural plasticity. Another study revealed that taVNS facilitates neural network reconstruction and functional compensation through multiple mechanisms: enhancing white matter remyelination, promoting angiogenesis, and inhibiting the TLR4/NF-κB and MAPK/NF-κB inflammatory signaling pathways (54). These effects collectively create a favorable microenvironment for axonal regeneration and synaptic plasticity.

### 3.5 Protecting the integrity of the blood–brain barrier

There exists a close interaction between the blood–brain barrier (BBB) and cerebral infarction (64). The BBB primarily maintains the homeostasis of the central nervous system. During cerebral infarction, ischemia and hypoxia lead to energy failure, triggering endothelial cell dysfunction (65). Concurrently, inflammatory factors activate matrix metalloproteinases, which degrade tight junction proteins and increase BBB permeability. Following reperfusion, oxidative stress and leukocyte infiltration further exacerbate BBB damage, resulting in vasogenic edema, hemorrhagic transformation, and aggravated neuroinflammation (66). BBB disruption not only worsens brain injury but also impacts treatment strategies, such as increasing the risk of bleeding after thrombolysis.

## 4 Current challenges and future directions

### 4.1 Lack of standardized stimulation parameters

The lack of consensus on optimal stimulation parameters remains a major barrier to clinical translation. Current studies use highly variable protocols, which not only complicate cross-study comparisons but also hinder the development of evidence-based guidelines. Existing studies demonstrate substantial variations in key parameter settings. For electrical stimulation parameters, frequencies range from 20 to 30 Hz—for instance, Zhang et al. (26) employed a 20/4 Hz dual-frequency approach while Li et al. (48) used a 30 Hz single-frequency protocol, with observed efficacy differences of 15–20%. Current intensities vary between 0.1–5 mA, pulse widths range from 0.1–1 ms, and treatment durations span from 10 days to 6 months. This lack of parameter standardization not only compromises comparability across studies but also creates a translational gap between preclinical and clinical research. Animal studies by Long et al. (54) utilizing 20 Hz/0.5 mA parameters contrast with clinical investigations by Wang et al. (30) using 25 Hz/0.1 mA, highlighting a significant barrier in translational medicine. Importantly, the clinical effects of stimulation parameters demonstrate multidimensional dependencies. Anatomically, individual variations in auricular nerve distribution affect targeting precision. Physiologically, work by De Ferrari and Schwartz (67) established that electrode spatial configuration, waveform characteristics, and timing parameters can selectively activate distinct neural pathways, yielding specific neuromodulatory effects.

To overcome these challenges, it is imperative to establish a three-tier standardization system: (1) A fundamental parameter framework to define core parameter ranges through multicenter adaptive RCTs; (2) A dynamic adjustment protocol developing closed-loop regulation devices based on multimodal feedback; and (3) Individualized titration standards, establishing stratified titration processes with reference to international consensus guidelines, thereby providing parameter benchmarks for precise neuromodulation.

### 4.2 Insufficient mechanistic research

Although animal studies have established that taVNS confers neuroprotection via cholinergic anti-inflammatory pathways, neurovascular remodeling, and synaptic plasticity mechanisms, a significant translational gap remains between these preclinical findings and clinical application. Current mechanistic research faces three major limitations. First, there are pathophysiological differences between standard animal models of stroke and human cerebrovascular disease, particularly in the complexity of cortico-striatal circuits, which may differentially influence taVNS responsiveness (68). Second, although some studies have explored the effects of taVNS on biomarkers such as heart rate variability, TMS parameters, pupillometry, neuroimaging, and event-related cortical potentials, research focused specifically on stroke populations remains relatively limited. Third, the relationship between stimulation parameters and underlying mechanisms remains unclear. Different frequencies may engage distinct neural circuits, yet no established correlations link specific parameters to their target pathways. This mechanistic fragmentation considerably hinders the development of precision therapies. Fourth, mechanisms such as anti-inflammatory effects, angiogenesis, and blood–brain barrier protection currently rely primarily on preclinical evidence for support. Although these mechanisms have been thoroughly validated in animal models (49, 54, 56), confirmatory human biomarker data remain considerably limited. For instance, while reductions in pro-inflammatory cytokine levels have been observed in human studies of non-stroke conditions (46), such evidence remains scarce in stroke populations. Similarly, angiogenesis and blood–brain barrier integrity have predominantly been demonstrated in rodent models (54, 57, 68), with no direct validation via imaging or biomarkers in human trials to date.

To address these challenges, future research should adopt a bidirectional translational framework. Basic studies should integrate more clinically relevant animal models—such as aged primates with metabolic comorbidities—combined with optogenetic-fMRI techniques to dynamically map taVNS-induced neural circuit modulation (69). Clinical studies should implement standardized, multimodal neuroimaging protocols through multicenter collaborations, along with biomarkers including heart rate variability, pupillometry, and event-related cortical potentials, to quantitatively evaluate taVNS therapeutic effects in stroke patients. Moreover, although preclinical evidence strongly supports anti-inflammatory effects mediated by the cholinergic pathway, clinical outcomes have been inconsistent. A recent systematic review and meta-analysis found no consistent evidence for an anti-inflammatory effect of VNS across human studies in various clinical conditions (70). This discrepancy underscores a notable translational gap and highlights the impact of variables such as study design, patient heterogeneity, stimulation parameters, and selection of inflammatory biomarkers. Incorporating these negative findings is essential for a balanced perspective and underscores the need for more rigorously controlled clinical trials specifically designed to investigate the anti-inflammatory mechanisms of taVNS in stroke populations.

### 4.3 Limitations in clinical study design

Current clinical studies on taVNS exhibit significant design limitations, primarily manifested in small sample sizes, short follow-up durations, inadequate blinding methodologies, and a lack of multicenter trials (Table 3). Most clinical trials to date have been limited by small sample sizes, which substantially reduces statistical power and generalizability of findings. For instance, while the study by Liu et al. included 80 participants, many others enrolled only 12–20 individuals, making it difficult to draw firm conclusions about efficacy (31). This insufficient statistical power makes it difficult to generalize conclusions. Additionally, some trials lack sham stimulation control groups or fail to implement double-blinding, making them susceptible to placebo effects. For example, although Wu et al.’s study adopted a randomized controlled design, it did not clearly describe blinding procedures, potentially compromising the reliability of results (24). Future research should conduct multicenter, large-scale randomized controlled trials with follow-up periods extending beyond 1 year to comprehensively evaluate the long-term efficacy and safety of taVNS. Moreover, strict adherence to CONSORT guidelines is essential to improve blinding methods and control group designs, ensuring the scientific validity and reliability of study outcomes.

**Table 3:** Key limitations of current clinical studies on taVNS in post-stroke rehabilitation.

Limitation category	Description	Examples from literature	Impact on evidence level
Small sample sizes	Majority of studies include fewer than 30 participants; underpowered to detect moderate effects	n = 14 (25); *n* = 20 (20); *n* = 12 (28)	Limited generalizability; increased risk of Type II errors
Heterogeneous protocols	Wide variability in stimulation parameters (frequency, intensity, pulse width, duration) and combined therapies	Frequencies: 20–30 Hz; Intensity: 0.1–3 mA; Pulse width: 0.1–1 ms; Duration: 10 days to 6 months	Difficult to compare outcomes; impedes protocol standardization
Short follow-up periods	Few studies include long-term follow-up (>6 months); most assess only immediate or short-term effects	15 days (24); 10 days (25); 6 months (31)	Sustainability of therapeutic effects remains unclear
Inadequate blinding	Some trials lack sham controls or do not fully implement double-blinding, increasing risk of placebo effects	Blinding method not clearly described; several studies use active sham but others use no stimulation (24)	Potential overestimation of treatment effects
Lack of multicenter trials	Almost all studies are single-center, limiting participant diversity and generalizability	All included studies are from single institutions	Results may not be replicable across different clinical settings
Individual variability	Anatomical and pathological differences (e.g., stroke type, lesion location) are rarely controlled or analyzed in subgroups	Better outcomes in ischemic vs. hemorrhagic stroke (29); variability in nerve anatomy (20)	Personalized application remains challenging
Lack of multicenter trials	Almost all studies are single-center.	All included studies are from single institutions.	Limits participant diversity and generalizability of findings.

### 4.4 Individual variability affects treatment efficacy

The therapeutic efficacy of transcutaneous auricular vagus nerve stimulation (taVNS) varies substantially across individuals, largely due to factors such as anatomical differences, stroke type, and disease stage (Table 3). A major source of variability lies in the natural diversity of vagus nerve branching patterns in the ear, which can influence stimulation precision and clinical outcomes, as noted by Badran et al. (20). Furthermore, the distinct pathophysiological features of ischemic versus hemorrhagic strokes contribute to differential responses to taVNS. For instance, Wang et al. (29) reported that ischemic stroke patients show more pronounced improvements in motor function compared to those with hemorrhagic strokes. To improve patient stratification, future studies should incorporate detailed subgroup analyses based on lesion location, severity, and chronicity of disease. In most clinical applications, taVNS is administered to the left ear. This preference stems from the fact that the right vagus nerve has a stronger influence on the sinoatrial node of the heart; excessive stimulation on the right side may therefore carry a higher risk of significant bradycardia or transient asystole. Nonetheless, some studies have explored right-ear or bilateral stimulation (13, 19, 20, 32, 49). We hypothesize that bilateral taVNS may concurrently activate vagal afferents from both ears, potentially leading to broader and more robust neuromodulatory effects. However, the underlying mechanisms and comparative therapeutic efficacy of these approaches remain to be thoroughly investigated.

Additionally, taVNS outcomes are influenced by a range of demographic, physiological, genetic, and clinical factors. Older age, for example, may be associated with reduced neuroplasticity and slower recovery, possibly attenuating response to taVNS (71). Age-related changes in autonomic tone and neurotransmitter systems may further modulate treatment effects. Sex differences—such as variations in autonomic function, inflammatory reactivity, and neuroendocrine pathways—also likely contribute to variability in responses (72). Baseline autonomic function is another key factor, as taVNS primarily modulates parasympathetic activity, and individuals with pre-existing autonomic dysfunction may exhibit differential responses (73). Genetic factors, including polymorphisms related to neuroinflammation, neuroplasticity, or cholinergic transmission, could also underlie interindividual variability (74). Finally, comorbidities such as diabetes, hypertension, or depression—which disrupt autonomic balance and neuroinflammatory pathways—may further influence taVNS efficacy. Together, these factors highlight the need for personalized taVNS protocols to optimize therapeutic outcomes.

### 4.5 Standardization challenges in devices and operational procedures

A major obstacle in current clinical applications of taVNS is the lack of unified standards for devices and operational protocols. Existing commercial devices exhibit significant deficiencies in standardized guidelines for home use, leading to considerable variability in parameter settings, treatment duration, and session frequency. Studies indicate that without standardized protocols, patient adherence and accuracy in self-administered therapy remain suboptimal, with some clinical trials reporting home treatment completion rates as low as 60–75% (31). Furthermore, current open-loop systems cannot adjust parameters in real-time based on individual physiological feedback, directly compromising treatment consistency. To address these issues, there is an urgent need to develop intelligent closed-loop systems that integrate real-time monitoring technologies with adaptive algorithms to enable dynamic parameter optimization. Concurrently, standardized user interfaces and operational workflows must be established. It is also critical to develop performance evaluation and quality control specifications tailored for home-use taVNS devices, referencing regulatory standards such as those set by the FDA for medical neuromodulation equipment.

## 5 Conclusion

TaVNS has emerged as a promising non-invasive neuromodulation therapy for post-stroke disorders, demonstrating efficacy in addressing motor dysfunction, depression, insomnia, dysphagia, cognitive impairment, and sensory deficits. By targeting the auricular branch of the vagus nerve, taVNS activates central pathways involved in neuroplasticity, anti-inflammation, angiogenesis, and blood–brain barrier protection, offering a multifaceted approach to stroke rehabilitation. Clinical and preclinical studies highlight its potential to enhance functional recovery and improve quality of life for stroke survivors, with advantages such as minimal side effects, high patient compliance, and suitability for long-term use.

However, several critical challenges persist, such as the absence of standardized stimulation protocols, incomplete elucidation of underlying mechanisms, methodological constraints in clinical trials, significant interindividual variability in treatment efficacy, and the pressing need for harmonized devices and procedures. Future research should prioritize large-scale, multicenter randomized controlled trials, bidirectional translational studies, and the development of closed-loop systems to optimize taVNS protocols. By addressing these gaps, taVNS could become a cornerstone of personalized post-stroke rehabilitation, bridging the gap between innovative neuromodulation and clinical practice.

In summary, taVNS represents a transformative therapeutic avenue for stroke recovery, with the potential to revolutionize neurorehabilitation through its non-invasive nature, broad applicability, and mechanistic versatility. Continued advancements in research and technology will be pivotal in unlocking its full clinical potential.
